# Study Protocol: Evaluation of a DVD intervention designed to meet the informaton needs of patients with head and neck cancer and their partner, carer and families

**DOI:** 10.1186/s12913-016-1875-7

**Published:** 2016-11-22

**Authors:** Vicki Parker, Leearna Bennett, Douglas Bellamy, Benjamin Britton, Sylvia Lambert

**Affiliations:** 1 Hunter New England Local Health District, New Lambton Heights, Australia; 2 University of Newcastle, Callaghan, Australia; 3 School of Health, University of New England, Armidale, NSW 2351 Australia; 4 Psycho-oncology, Calvary Mater Hospital, Waratah, Australia; 5 Ingram School of Nursing, McGill University, Montreal, Canada

## Abstract

**Background:**

Patients who undergo surgery for cancer of the head and neck and their families face complex and difficult challenges and are at risk of anxiety and depression and inability to cope with symptom and treatment burden. Information available to support them is not flexible enough to adjust to individual need.

**Design/Methods:**

A randomised clinical trial pre and post intervention design, comparing the use of a tailored DVD intervention, provided preoperatively and used throughout the post- operative period, with usual treatment. One hundred fifty-six individuals or partner couples will be randomly recruited into either the intervention or control group. A survey will be administered at three time points, preoperatively, post operatively and 3 months post-surgery. Anxiety and empowerment are the primary outcome measures. Qualitative data about use of the resource will be gathered by phone interview.

**Discussion:**

This is the first study to rigorously evaluate the impact of a DVD intervention for this group of patients and their family members. The study will help to understand the impact of information usage on patient and family well- being and test a means by which to evaluate information and education resources for this and other cancer patient groups.

**Trial registration:**

ACTRN12614001104640. Date registered: 17/10/2014.

## Background

Head and neck cancer has a global burden of more than 550,000 cases annually [1]. Incidence rates vary between countries due to increased rates with tobacco epidemics currently peaking in some regions while declining in others where tobacco use has peaked and is falling, and with the increasing incidence associated with human papillomavirus infection [2]. Head and neck cancers diagnosed in Australia in 2009 (3896) accounted for 3.4 % of all cancers diagnosed (114,137), with the number of head and neck cancers diagnosed increasing between 1982 (2475) to 2009 (3896). This increase is attributed to an increasing aging population [3].

The treatment for head and neck cancer is often drastic and debilitating, with surgery effecting vital functions such as swallowing and breathing, as well as speech. This frequently results in poorer patient outcomes in terms of quality of life [4] that have long term impact and require significant lifestyle and psychological adjustment by patients and families [5].

Evidence points to the need for significant and broad ranging support for this group of patients who can often be overlooked in large scale cancer support programs [6]. In many cases, the complexity of the physical and functional impairments resulting from head and neck cancer or the treatment can lead to high levels of anxiety, impacting negatively on self-image, self-efficacy [7] social isolation, and depression [8]. Holland and Weiss [9] have recommended that psychosocial aspects should be integrated into routine cancer care for these patients throughout their illness trajectory. Provision of information to cancer patients has been shown to reduce anxiety and support decision making [10]. However the amount of evidence available to support information interventions is small and limited by poor quality and lack of attention to practical implementation [11].

The availability, content and quality of written information specific to the individual needs of patients with head and neck cancer have been repeatedly identified as areas requiring attention [12, 13].^,^ It is well-documented that patients require personalized information to be able to participate optimally in decision-making and self-care and that information giving needs to be informed by the preferences of patients and families and matched to significant events that occur across the illness trajectory [5].

The role of partners and family members in supporting cancer patients is also recognised as central to patient well- being [10]. They may also be impacted by the burden of care and treatment [14], and hence require timely targeted information that increases their understanding and capacity to contribute to care. Carers are also at risk of psychological distress, with reports of up to 70 % of carers of cancer patients experiencing depression and 39 % reporting anxiety [15, 16]. A recent review identified that some partners and family caregivers’ most prominent needs pertain to curtailing the impact of their roles and responsibilities on daily living and information (e.g., what to expect) [17].

A recent review of online interventions for cancer patients finds that the majority of evaluations of information related interventions focus on patient usage and satisfaction, with few reporting quantifiable clinical outcomes [18]. Additionally, there are very few studies reporting specifically on the impact on clinical outcomes. For example, [19] report on a web-based psycho-educational program for cancer patients and their caregivers that delivered a post-intervention decrease in emotional distress, and improvements in quality of life indicators. Similarly, a Korean internet-based cancer-related fatigue program, reports significant improvements in fatigue, quality of life, and anxiety for the intervention group [20]. Another program that provided head and neck cancer patients with educational resources, the ability to engage with other patients via an online forum, and an at-home monitoring system, found a significant improvement in quality of life indicators after 6 weeks compared to the control group, although this difference was not sustained after 6 months [21]. However, given the design of this study, the effect of providing educational resources alone could not be assessed. While internet-based educational programs for cancer patients certainly offer promise, variable skills in technology use have been highlighted as a confounding factor in the assessment of such intervention programs, particularly for an older cohort [18]. An Australian intervention providing prospective chemotherapy patients with a DVD preparing them for treatment and possible side effects, found benefits in improving patients’ confidence in coping with cancer, although it did not reduce pre-treatment anxiety. Nonetheless, the authors reinforce the need to provide patients with informational resources such as DVDs, as an adjunct to education provided directly by clinicians, given increasing challenges resulting from workforce shortages and the greater emphasis on ambulatory care [22].

In response to clinicians’ concerns regarding the unmet needs of patients who undergo surgery for head and neck cancers we undertook a qualitative study to identify the needs of this group of patients and their family members [5]. An important finding from this study was participant’s reports of the inadequacy of information provided to them and the difficulties they had understanding and using information effectively. Information is often developed for or by health professionals and hence may use language not easily understood by patients and their families. The needs identified included the need to feel in control and to have information about what to expect at various stages. Concerns varied according to the tumour site and the nature of surgery and any adjuvant therapies. Because of the many and varied types of surgery it was not surprising that very few people felt they had information specific to their needs [5]. These finding supports research that highlights the importance of accurate, comprehendible and timely information pre-surgery and post-surgery for head and neck cancer patients [23, 24]. At present there appears to be no systematic way for patients and partners to gain information at the various stages across the surgical journey and in accordance with their changing needs. The study described in this protocol aims to evaluate a DVD resource produced for this purpose.

## Methods/Design

### Study design

A multicentre randomised pre and post intervention design controlled trial (RCT) testing the effectiveness of a DVD information resource intervention. The overall aim of this study is to evaluate impact on mood, coping and self- efficacy, together with the utility of the first DVD education intervention designed to meet the information needs of head and neck cancer patients who undergo surgery in Australia and their partners and family members.

The primary hypothesis is: Patients and carers in the intervention group will report lower anxiety post-op and 3 months post-baseline compared to those in the control group.

The secondary hypothesis is: Patients and carers in the intervention group will report lower depression and symptom distress, fewer unmet information needs, and feel better prepared for the various steps in their treatment compared to those in the control group.

The study was funded by a Hunter New England Health District Innovation Support Grant.

### Setting

Two large metropolitan and one large regional hospital across two States in Australia. Patients recently diagnosed with a cancer of the head and neck and who are referred for surgery, and their nominated partner, carer or family member will be recruited from participating sites where surgery for Head and Neck cancers is conducted.

### Participants

Participant inclusion criteria are:

- Group 1. Adults aged 18 and over who have been diagnosed with a primary, early-stage head and neck cancer, whose treatment plan includes surgery.
- Group 2. Partners or family members aged 18 and over involved in the care and support of patient participants from Group 1.

Participants will need sufficient fluency in English and be able to participate in the study. While the aim is to recruit dyads where possible, consenting patients whose partner, carer or family member does not consent to participate, will still be eligible.

In accordance with our sample size calculation 156 (78 in in the intervention group and 78 in the control group) will be recruited into the study. If they have a partner, carer or family member who is also willing to participate they will be recruited and provide ‘linked’ data to the patient’s responses. Therefore we aim to recruit 156 individual patients or patient couples (total 156 to ≤312 individuals). The probability is 80 % that the study will detect a minimum important difference of 1.5 on HADS scores at a one-sided 0.05 significance level.. This is based on the assumption that the standard deviation of the response variable is 3.75

#### Recruitment

Clinicians will identify patients meeting the inclusion criteria during their initial specialist appointment or multidisciplinary team meeting (MDT) appointment. The surgeon or care coordinator will introduce eligible patients to the study and give interested participants a study pack, including study information and a consent form and a similar study pack to pass on to their partner, carer or family member. A Clinical Nurse Consultant (CNC) will consent the patient to the study and conduct the baseline survey at the pre-operative clinic. If the partner, carer or family member is not present, the baseline survey will be posted with a reply paid envelope to be completed at home. Following this, the CNC will apply the outcome of randomisation and the participant will be allocated to treatment as usual or the intervention group.

#### Randomisation

A randomisation schedule will be established for each site using the website Randomization.com (http://www.randomization.com) to generate block randomised allocation to either the treatment or control using random block sizes. The schedule concealment will be maintained by a researcher at the central site who will not be involved with recruiting the patients to ensure allocation is concealed. Once participants are identified as eligible for the study, the recruiter will contact the researcher by phone or email to register the participant to the trial, including assigning the participant a unique study ID and allocating them to one of the conditions as per the randomisation schedule. Participants will not be blinded as this is a trial of a resource in addition to usual treatment, as such participants will be aware if they received the intervention DVD or not. If patients don’t complete the baseline survey, they will not be randomised to the trial. Patients will mostly use the study intervention at home, hence the chance of control participants being exposed to the intervention is minimal.

### Intervention

The intervention involves a DVD for patients and their partners integrated into pre and post-surgical care. Participants will receive the education resource, in the form of a DVD, pre operatively, usually in the pre op clinic, as well as information provided as usual care. Each dyad (patient and partner) will receive one DVD which they may use individually or together.

**Fig. 1:**
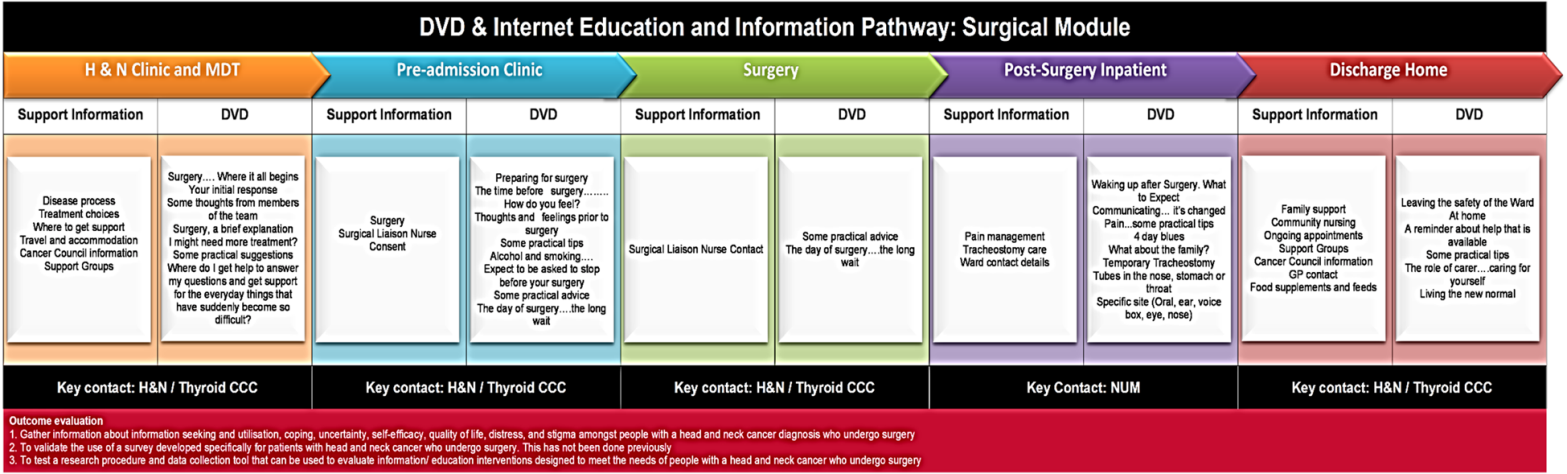
DVD Delivery Pathway

The DVD comprises a series of chapters in which key issues and events are addressed through conversations with patients and staff (Fig. 1). The purpose of the DVD is to provide information and opportunity for patients and families to have their questions and concerns addressed as they progress from surgery through to recovery or on to adjunctive therapies. The DVD is designed in a way that participants can tailor what they learn, how often they engage with the DVD and its associated resources and how much support they seek in working with the resource.

The actors in the DVD are real patients who have undergone surgery for removal of cancers from various head and neck sites, and health professionals (doctors, nurses, allied health) who patients will encounter during their journey.

#### Usual care

Usual care patients will have access to the standard resources and services currently available to patients undergoing surgery for head and neck cancer. This includes the [25].

### Data collection

Data will be collected using a self-administered survey (one for patients and one for partners, carers or family members) at three time points i.e.,; baseline (prior to surgery), post-surgery (usually at follow up clinic) and 3 months post-baseline. Information about how the resource is being used and who is using it will also be collected via phone call at T2.

**Fig. 2:**
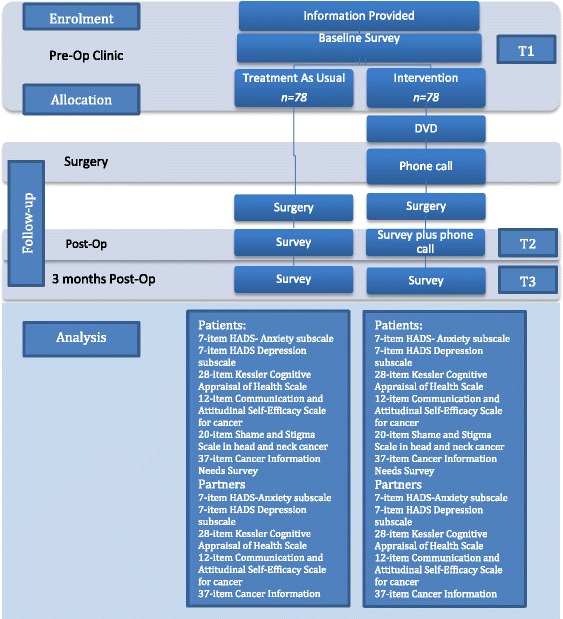
Study timeline

Those participants randomised to the intervention group will receive the intervention DVD to take home and a follow-up phone call will be made with a week to ensure they are clear about how to use the DVD. Follow up surveys will be conducted at the post-op clinic visit and again at 3 months post-surgery. At time point 2 participants will again be contacted by phone to discuss how the resource is being used and to identify any concerns that may have arisen. A standardised discussion guide with questions regarding the DVD will be used and handwritten notes taken. The 3 month follow up may be conducted via post, telephone or web survey (Fig. 2).

### The survey tool

The survey tool comprises a number of validated tools that have been used widely in patients who have had treatment for cancer. Demographic data such as age, postcode, marital status, income and level of education along with general health information will also be collected. Prior to commencement of the study the survey was piloted with 11 patients 6 family members and modified according to feedback. The original survey was reduced in size as patients reported it was too time consuming to complete, particularly in the clinic context.

Table 1 summarises all variables that will be measured and psychometric properties of the measures that will be used, the primary and secondary outcomes and moderators that will be surveyed, including the chosen measures. Outcome selection was based on Lazarus and Folkman’s (1984) Stress and Coping framework [26], where outcomes either characterise the coping process (appraisal, dyadic and individual coping strategies, and self-efficacy) or indicate the extent to which the coping process was successful in addressing stressors (mental health and well-being outcomes: anxiety, cancer distress, depression, quality of life, and relationship satisfaction). Anxiety and empowerment are the primary outcomes, as the DVD intervention focuses on coping strategies to manage high levels of anxiety directly and/or cope with cancer demands known to trigger anxiety. The degree to which participants feel challenged and supported at each time point will also be measured using a five point Likert scale.

**Table 1:** Outcome measures

Outcomes	Measures and psychometrics	
	Patients	Partners
Anxiety	7-item HADS-Anxiety subscale [28] (α = .68−.93)	
Depression	7-item HADS Depression subscale [28] (α = .68−.93)	
Appraisal	28−item Kessler Cognitive Appraisal of Health Scale [29] (α > .70)	28-item Kessler Cognitive Appraisal of Health Scale [29] [adapted] (α > .70)
Self-efficacy and empowerment	12-item Communication and Attitudinal Self-Efficacy Scale for cancer [30] (CASE-Cancer; α = .76−.77) 29-item Strategies Used by People to Promote Health (SUPPH, α = .76−.92 [31] 10-item Perceived Efficacy in Patient-Physician Interactions (PEPPI, α = .91) [32]	12-item Communication and Attitudinal Self-Efficacy Scale for cancer [30] (CASE-Cancer[adapted]; α = .76−.77) 29-item Strategies Used by People to Promote Health (SUPPH, α = .76−.92) 48-item Caregiver Empowerment Scale (α = .76−.92) 10-item Perceived Efficacy in Patient-Physician Interactions (PEPPI, α = .91)
Shame and Stigma Scale in head and neck cancer	20-item Shame and Stigma Scale in head and neck cancer [33]	
Information needs	37-item Cancer Information Needs Survey [34]	

### Data analysis

Intention-to-treat and per protocol analysis will be conducted. The primary outcome, anxiety will be measured repeatedly across the three time periods and, therefore, analysed using generalised linear mixed models (GLMM). In this context, the GLMMs are similar to linear regression models, but take account of the correlation between repeated measurements on individuals. Their advantage over a traditional repeated measures analysis of variance is that they use all available data under the assumption that missing data are missing at random. Sensitivity analysis will explore the robustness of this assumption. GLMM will also be used to explore the secondary outcome measures. The study is powered on a sample of individual patients. Partners were outside the calculation and are a secondary outcome only.

Qualitative data collected at phone interview (or face to face if possible) at time point 2 will be transcribed, coded and themed to produce a descriptive account of participants’ views about the resource and experiences of using the DVD resource.

### Ethical considerations

The study was approved by Local Health District Research Ethics and University Committees, and registered with the NSW Clinical Trials register. The study will be conducted in accordance with the NHMRC ethical guidelines. Potential participants will be receive an information letter outlining what will be expected of them, informing them of their right to withdraw at any time and guaranteeing their privacy and anonymity and that participation will not impact deleteriously on the care they will receive.

The study was developed in accordance with the Medical Research Council framework for developing and evaluating complex interventions and CONSORT guidelines.

## Discussion

Although blinding is not possible as participants will be aware if they received the intervention DVD or not, it is anticipated that contamination will be minimized because participants will be mainly using the study intervention at home. A particular difficulty for the study is the low numbers of patients who undergo surgery and hence it may take time to get the required numbers.

Meeting the information needs of patients who undergo surgery for head and neck cancers and their families is challenging due to the diversity of tumour site, nature and degree of surgical intervention and unique personal characteristics mean that needs differ from person to person. Further, needs vary over time as confidence levels change and new challenges are encountered.

Improvements in models of care which including the introduction of MDT meetings [27] have increased the opportunities for communication and information provision. However, appointment times are often short and information may not always presented in a way that helps patients and families consider what the experience will mean for them [5]. It is anticipated that using the DVD to inform conversations will help to reduce some of these barriers, helping patients to anticipate and be prepared for the challenges they will face and to gather and strengthen their support networks. Partners and family members’ needs may differ from those of the patient and meeting their needs also contributes to positive outcomes for patients [17]. While some needs have been shown to be similar to those of cancer patients, they may differ in detail and perspective [10] Partners and family members report being more concerned with non-medical topics such as coping with cancer and impact on relationships. The systematic review conducted by [10] highlighted the lack of studies reviewing the information needs of partners and family members in relation to cancers other than breast and prostate. Although not specific to head and neck cancer this review highlights the importance of monitoring needs of both groups and ensuring information is provided that supports the patient and their supporting network.

Little empirical evidence exists that compares means of providing information for patients and partners. The findings of this study will provide evidence that will improve the provision and use of information for patients and their families. It will highlight the role of timely salient information provision in improving patient and carer outcomes after surgery by demonstrating if and how the intervention leads to their increased capacity to cope with and manage their care. In particular it will indicate the impact of the intervention on psychological well-being. It will also demonstrate the effectiveness of an approach to evaluating the use of resources by patients and families. It is anticipated that following evaluation the DVD and accompanying resources will be made widely available for patient benefit nationally and internationally.

## Abbreviations

CNC: Clinical Nurse Consultant
GLMM: Generalised linear mixed models
MINT: Meeting information needs together
RCT: Randomised controlled trial
